# Trauma Training Courses and Programs in Low- and Lower Middle-Income Countries: A Scoping Review

**DOI:** 10.1007/s00268-021-06283-1

**Published:** 2021-09-05

**Authors:** Rachel J. Livergant, Selina Demetrick, Xenia Cravetchi, Janice Y. Kung, Emilie Joos, Harvey G. Hawes, Abdullah Saleh

**Affiliations:** 1 Office of Global Surgery, Department of Surgery, University of Alberta, Walter Mackenzie Health Sciences Centre, 2D2.238440 - 112 Ave NW, Edmonton, AB T6G 2B7 Canada; 2 John W. Scott Health Sciences Library, University of Alberta, Walter C. Mackenzie Health Sciences Centre, 2K3.288440 - 112 Ave NW, Edmonton, AB T6G 2R7 Canada; 3 Division of General Surgery, Trauma and Acute Care Surgery, Vancouver General Hospital, University of British Columbia, Jim Pattison Pavilion, 899 W 12th Ave, Vancouver, BC V5Z 1M9 Canada

## Abstract

**Background:**

Injury is the leading cause of morbidity and mortality in low- and lower middle-income countries (LMICs). Trauma training is a cost-effective way to improve injury outcomes. Several trauma programs have been implemented in LMICs; however, their scope and effectiveness remain unclear. In this review, we sought to describe and assess the current state of trauma training in LMICs.

**Methods:**

We searched MEDLINE, Embase, Global Health, Cochrane Library, and ProQuest Dissertations & Theses Global for trauma training courses in LMICs. An additional gray literature search was conducted on university, governmental, and non- governmental organizations’ websites to identify trauma-related postgraduate medical education (PGME) opportunities.

**Results:**

Most studies occurred in sub-Saharan Africa and participants were primarily physicians/surgeons, medical students/residents, and nurses. General and surgical trauma management courses were most common, followed by orthopedic trauma or plastic surgery trauma/burn care courses. 32/45 studies reported on participant knowledge and skills, 27 of which had minimal follow-up. Of the four studies commenting on cost of courses, only one demonstrated cost-effectiveness. Three articles evaluated post-course effects on patient outcomes, two of which failed to demonstrate significant improvements. Overall, 43.0% of LMICs have PGME programs with defined trauma competency requirements.

**Conclusions:**

Current studies on trauma training in LMICs do not clearly demonstrate sustainability, cost-effectiveness, nor improved outcomes. Trauma training programs should be in response to a need, championed locally, and work within a cohesive system to demonstrate concrete benefits. We recommend standardized and contextualized trauma training with recertifications in LMICs for lasting and improved trauma care.

**Supplementary Information:**

The online version contains supplementary material available at 10.1007/s00268-021-06283-1.

## Introduction

Trauma and injury represent one of the greatest causes of mortality and morbidity globally, more than tuberculosis, HIV and malaria combined [1, 2]. Despite a decreasing trend in injury-linked disability-associated life years (DALYs) worldwide, low- and lower middle-income countries (LMICs) continue to shoulder a disproportionate burden of traumatic incidents.

Limitations to appropriate trauma care and management in LMICs are multifactorial. After a lack of equipment, inadequate training and education of healthcare providers (HCPs) are the most common barrier to effective trauma care in these settings [3]. Several trauma courses have been implemented in LMICs including Advanced Trauma Life Support (ATLS); Primary Trauma Care (PTC); and Trauma Team Training (TTT).

ATLS is considered the gold standard for trauma training globally; however, the program has faced criticism for being too costly and uncontextualized for low-resource settings [4, 5]. It was due to the lack of accessibility of ATLS that courses like PTC and TTT emerged. Although PTC and TTT train multidisciplinary teams, unlike ATLS which centers around surgical staff, these courses are narrower in their scope of traumatology topics. Consequently, although these courses have addressed some of the criticism faced by ATLS, there is still a debate as to their effectiveness to meaningfully reduce injury-related morbidity and mortality [6, 7].

Another layer of trauma care education in LMICs is postgraduate medical education (PGME) programs, many with specific trauma competencies built into the curricula [8, 9]. The restructuring and increased development of these PGME programs in LMICs are promising for the field of traumatology; however, there lacks a description of available PGME programs, limiting the ability to comprehensively evaluate trauma training systems in LMICs.

To date, there remains uncertainty as to the extent and effectiveness of trauma training in LMICs. Notably, there is no consensus as to what is the most successful, appropriate, and cost-effective program for definitive hospital care trauma training. This scoping review aims to examine the current ecosystem of trauma training in LMICs, with a specific emphasis on the effectiveness, breadth, multi-disciplinarity, and sustainability of these programs.

## Methods

This scoping review was registered in Open Science Framework (OSF, osf.io/2q9d3) and reported in accordance with Preferred Reporting Items for Systematic Review and Meta-Analysis-Extension for Scoping Reviews (PRISMA-ScR) framework (Online Resource 1) [10].

### Search strategy

Comprehensive searches were undertaken in Ovid MEDLINE, Ovid Embase, Ovid Global Health, Cochrane Library, and ProQuest Dissertations & Theses Global on Aug 24, 2020, using relevant keywords (Online Resource 2). Since there were major developments in global trauma training over the last 20 years, the searches were limited to articles published from 2000 and onward [11]. Gray literature, reference lists of reviews and retrieved articles, and consultations with experts were also conducted to identify additional studies.

### Study and program selection

Two reviewers independently screened titles, abstracts, and full texts. Studies were included if they were experimental or observational studies and excluded if they were book chapters, conference abstracts, or non-peer reviewed. Articles pertaining to HCPs, including but not limited to physicians, surgeons, medical officers, nurses, and medical students were included, while those describing laypeople or pre-hospital care providers were excluded. All trauma courses training that focused on trauma including the management of specific injuries such as orthopedic injuries, burns, traumatic brain injuries, and craniomaxillofacial injuries were included. Studies reporting educational programs focused exclusively on ultrasound, telemedicine, triage, trauma registries, critical care, and emergency medicine (including obstetrics, neonatology, and infectious diseases) were excluded.

### Data extraction and analysis

The following information was extracted from individual studies using a standardized extraction form: publication year, study setting, characteristics of trainees, course details, and course outcomes. Data from primary studies were extracted by one reviewer and independently verified by a second reviewer. Any discrepancies were resolved by consensus.

Study characteristics were analyzed and presented using a mixed-methods approach. The heterogeneous nature of this review precluded a meta-analysis. Instead, a narrative synthesis of results was undertaken using evidence tables and figures to aid in data presentation, when appropriate.

Outcomes included course effectiveness, suitability, sustainability, and breadth of topics and training. Course effectiveness was defined based on changes to patient morbidity and mortality or skills of participants. Suitability refers to the extent to which courses were contextualized to the setting and engaged local stakeholders during implementation. Sustainability reflects the longevity of the course in terms of follow-up, recertification processes and cost-effectiveness, while course breadth refers to the variety of types of HCP participants and trauma topics.

### Gray literature search for PGME programs

A gray literature search for surgical PGME programs with trauma components in LMICs was undertaken. Accredited programs such as residency, fellowship, diploma, certificate, Masters, and Doctoral programs were used as search terms, identified, and included for consideration. PGME programs were reviewed for trauma components and duration.

## Results

### Trauma care training courses

**Fig. 1:**
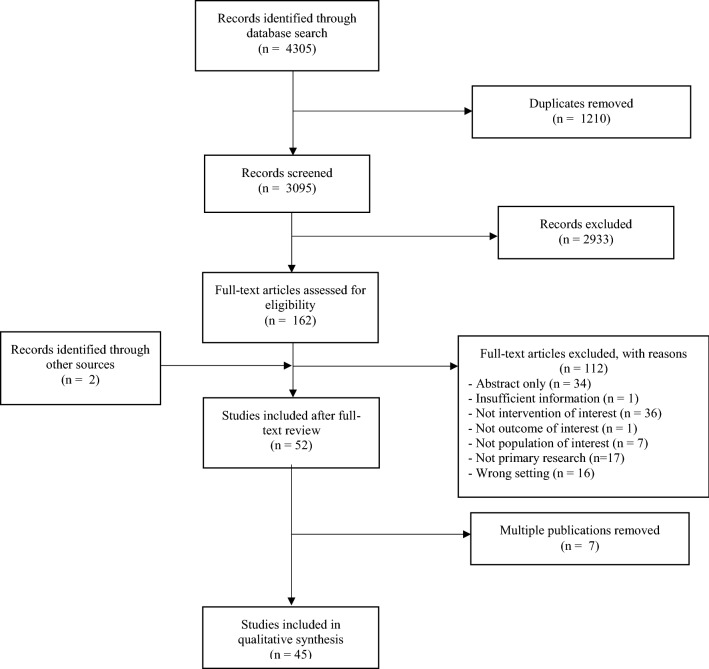
The literature search and study selection flowchart

We retrieved 4305 articles from the electronic search, of which 3095 underwent title and abstract screening. Seven studies were multiple publications reporting on the same training course or study cohort and therefore are reported with their parent study to avoid duplication, resulting in 45 unique studies (Fig. 1) [12–63]. Descriptions of included studies can be found in Table 1.

**Table 1:** Description of trauma training courses in low- and lower middle-income countries identified from electronic database search

First author, year	Country	Program	Participants (n)	Outcome(s)
*East Asia and Pacific*				
Van Heng, 2008 [12]	Cambodia	“Surgical trauma training for non-surgeons” in rural hospitals	Physicians (2) Medical officers (19)	Increased overall confidence and skills managing trauma after training compared to before training based on scoring from instructors
Tajsic, 2017 [13]	Cambodia	Open Fracture Training Course (including post-traumatic defects)	Surgeons (112)	Significant improvement of theoretical understanding, technical skills and self-confidence in independent management of open fractures and reported a 70% primary healing rate of fractures
Kornfeld, 2019 [14]	Mongolia	Advanced Trauma Life Support (ATLS)	Physicians	Annual cost of ATLS with Mongolian instructors would be USD 10,709 compared to USD 68,038 if foreign instructors are required every year
Richard, 2009 [15]	Myanmar	Trauma Management Program (TMP)	Village health workers (300)	Majority of patients treated by trainees survived (91%). Unstructured interviews revealed that training during workshops was utilized in the field for trauma care
Yu, 2017 [16]	Solomon Islands	Pediatric Acute Care Training Course	Physicians (57)	Participants reported a mean 4.81/5 overall satisfaction with the course, but the course needed further sociocultural modifications. Post-course test results increased from 69.6% to 77.6%
Lam, 2018 [17]	Vietnam	Burn Management Training	Physicians (174) Nurses (131)	Participants significantly increased their knowledge scores on burn emergency management (p < 0.01) and mass burn injury management (p < 0.001) after participation in the course
Choi, 2020 [18]	Vietnam	Orthopedic Trauma Care Capacity Strengthening Training Workshop	Surgeons (19) Rehabilitation therapists (6) Nurses (2)	The orthopedic training fellowship workshop on trauma care received high satisfaction ratings from participants in terms of satisfaction of teaching tools (4.28/5); perceived benefit of course (4.17/5) and overall quality of the workshop (4.32/5)
*Latin America & Caribbean*				
Cioè-Peña, 2017 and Dickason, 2017 [19, 20]	El Salvador	Primary Trauma Care (PTC)	Physicians and nurses (18)	Overall critical skills in trauma management, time to operating room and mortality rates of trauma patients were not significantly altered by PTC. Number of FAST exams increased from 9.5% (pre-training) to 21.4% post-training (p = 0.04)
Carlson, 2012 [21]	Haiti	Orthopedic Trauma Care Specialist (OTCS) Residency Program	Surgical residents	Orthopedic trauma care specialists care versus traditional care resulted in 12,213 DALYs averted and cost savings of USD 134/DALY for every resident trained
Normore, 2017 [22]	Haiti	Orthopedic Trauma Symposium (OTS)	Medical students. (29) Residents (21) Physicians (9) Other (11)	Participants were satisfied with the level of difficulty (3.59), organization of topics (4.09), usefulness of exercises and helpfulness of instructional aids (4.71) of the OTS based on a five-point Likert scale
Kurdin, 2018 [23]	Haiti	Trauma Evaluation and Management (TEAM)	Physicians (32) Nurses (22) EMTs (10) Medical students (5)	Survey responses indicated that the course was satisfactory but needed adjustments for LMICs to include unavailability of specialists and increased nursing responsibilities
Pringle, 2015 [24]	Nicaragua	“Short trauma course with low cost simulation”	Physicians (18) Residents (15)	Written simulation scores improved from pre- to post- course (test increase by 15.4%, p < 0.001 and simulation by 33.6%, p < 0.001). Primary survey and c-spine immobilization times during simulation also improved
*Middle East & North Africa*				
El-Shinawi, 2015 [25]	Egypt	Sequential Trauma Emergency/Education ProgramS (STEPS)	Physicians, Surgeons, Residents, Laypeople (639)	Course demonstrated the sustainability of a trauma course transitioned from a high-income country to the LMIC over seven years
Alwawi, 2019 [26]	West Bank	Primary Trauma Care (PTC)	Physicians and Medical Students (445) Nurses (130) Midwives (11)	Increased knowledge and understanding of trauma management skills post-training (15.76/20) compared to pre-training (9.43/20) (p < 0.001)
*South Asia*				
Biswas, 2017 [27]	Bangladesh	Emergency Management of Severe Burns (EMSB)	Physicians (38)	Self-reported surveys indicate 43.8% and 31.2% of participants felt more confident in their overall burn management skills and handling of critical care burns, respectively
Tchorz, 2007 [28]	India	“Intensive trauma course”	Residents (23) Physicians and Surgeons (9)	Participants had significantly improved mean scores from pre-course (70.7%) to post-course (87.5%) trauma management knowledge tests (p < 0.001)
Light, 2009 [29]	India	American Burn Association/Children's Burn Foundation (ABA/CBF) Team Training	Healthcare practitioners (60)	Study reports improved nursing satisfactions, patient tolerance, burn management knowledge, earlier excision, and grafting and decreased antibiotic use post-intervention
Douglas, 2010 [30]	India	Advanced Trauma Life Support (ATLS)	Medical officers (Indian = 77; Australian = 44)	Trauma knowledge quiz scores demonstrated that the net pass rate for those who completed ATLS was 97% compared to 15% for those who did not take ATLS
Ali, 2015 [31]	India	Rural Trauma Team Development Course (RTTDC)	Healthcare providers (23)	Participants improved their trauma management knowledge and understanding (pre-test = 6.8; post-test = 13.6, p < 0.001) after the RTTDC. Additionally, the course had a high participant satisfaction level
O’Reilly, 2009 and 2011 [32, 33]	India and Sri Lanka	Alfred Trauma Team Training Program	Nurses (14) Physicians (12)	Participants demonstrated an improvement on team-based trauma scenarios, multiple choice tests, and written evaluations
Shrestha, 2018 [34]	Nepal	Emergency Room Trauma Course (ERTC)	Medical Students (97)	Participant MCQ knowledge scores improved from 64.2% to 89.8% (p < 0.001) and skills scores on OSCEs improved from 33.2% to 78.6% (p < 0.001)
Jawaid, 2013 [35]	Pakistan	Primary Trauma Care (PTC)	Physicians, Surgeons, Residents and Medical students (20)	Participants demonstrated increased trauma management skills (pre-test = 3.5/20, post-test = 9.5/20, p < 0.0001) and knowledge (pre-test = 19.5/30, post-test = 25/30, p < 0.0001) after the PTC course. Although 95% of participants scored > 70% on the knowledge test, only 20% of participants scored > 70% on the skills portion post-course
Sadiq, 2018 [36]	Pakistan	Primary Trauma Care (PTC)	Medical students (77)	Participants scored on average 5.104 points higher on their post-course trauma management quizzes compared to their pre-course quizzes (p < 0.001)
Soomro, 2020 [37]	Pakistan	Trauma Evaluation and Management (TEAM)	Medical students (294)	The course had a high satisfaction rate. With > 85% of participants indicating usefulness of the course in future practice and > 80% of faculty wishing to be involved in TEAM instruction. As well, there was a significant increase in trauma management knowledge for groups receiving TEAM training versus those without the course (p < 0.001)
*Sub-Saharan Africa*				
Ologunde, 2017 and Peter, 2015 [38, 39]	Burundi, Ethiopia, Kenya, Malawi, Mozambique, Rwanda, Tanzania, Uganda, Zambia and Zimbabwe	Primary Trauma Care (PTC)	Physicians (253) Nurses (98) Medical students (44) Clinical officers (40)	Six months after training, 92.7% of respondents indicated improved trauma management, with 52.8% using a systematic or ABCDE approach. Departmental changes to trauma care were mainly moderate (30%) with 23% of respondents indicating no change. Only 24.8% of respondents perceived improvement in trauma patient mortality and morbidity
Nogaro, 2015 [40]	Ethiopia, Kenya, Malawi, Mozambique, Rwanda, Uganda, Zimbabwe	Primary Trauma Care (PTC)	Physicians and Surgeons (240) Other healthcare practitioners (105)	91% of candidates showed a significant improvement in knowledge after the PTC course, with a median improvement of 17% (p < 0.05). As well, candidates’ confidence in trauma management improved by 20%
MacLeod, 2009 and 2011 [41, 42]	Kenya, Zambia	Acute Trauma Care and Fundamental Critical Care Support Course (ATC/FCCS)	Medical officers (27) Physicians and Surgeons (21) Nurses (14) Clinical officers (13)	Participants had increased overall trauma knowledge from an average of 51% to 63% (p = 0.002) post-course. Additionally, participants reported increased confidence over all 22 presented clinical trauma scenarios and 15 procedures
O’Sullivan, 2012 [43]	Malawi, Tanzania, Zambia	Global Emergency Care Skills (GECS)	Physicians (80) Nurses (5) Clinical officers (2)	Trauma management knowledge test scores were significantly improved from pre-course (58.71%) to post-course (78.26%) (p = 0.0001) for participants from all countries
Shaye, 2018 [44]	Rwanda, Zimbabwe	Craniomaxillofacial Trauma Course “Essentials in Facial Injuries” Course	Residents (12) Surgeons (5) Unknown/Other (6)	Mean pre-course and post-course test scores increased in both cohorts (cohort 1 = 30% increase, cohort 2 = 12% increase)
Tolppa, 2020 [45]	Democratic Republic of the Congo	Primary Trauma Care (PTC)	Physicians (36) and Nurses (23)	Although increased post-course test scores were maintained over two years, confidence skills significantly decreased (p = 0.03). As well, 36 participants indicated a lack of equipment availability, while 52 felt different procedures were required for managing local patients
Mock, 2005 and Quansah, 2008 [46, 47]	Ghana	Kwame Nkrumah University of Science and Technology (KNUST) Trauma Course for Rural Hospitals	Physicians (83)	Participants had significant increases in post-course scores and the majority felt trauma care practice had improved at one-year follow-up. Recommendations for improvement included more practical sessions, focus on orthopedics, and longer course duration
Berndtson, 2019 [48]	Ghana	Trauma Evaluation and Management (TEAM)	Medical students (62)	TEAM training resulted in higher post-course scores (69.1%) compared to pre-course (44.2%) scores (p < 0.001). Ghanaian medical students indicated infrastructure, teamwork issues and lack of physical equipment as major barriers to trauma care
Wanjiku, 2017 [49]	Kenya	“Emergency trauma course”	Medical students (22)	Immediately post-course, test scores and simulation scores increased by a mean of 15.5% and 45.5%, respectively (p = 0.0001). At nine months follow-up, simulation, knowledge and confidence scores had not significantly changed
Hill, 2018 [50]	Kenya	Trauma Evaluation and Management (TEAM)	Medical students (61)	TEAM training resulted in higher post-course scores (72%) compared to pre-course (57%) scores (p < 0.001)
Young, 2016 [51]	Malawi	Postgraduate Medical Education for Surgical Trauma	Residents (12)	The study reported on the creation of cohesive, national surgical residency programs with training in trauma care. Amputations significantly decreased in this period, while limb saving surgeries increased
Petroze, 2015 [52]	Rwanda	Advanced Trauma Life Support (ATLS) and Trauma Team Training (TTT)	Surgeons, Nurses, Residents (64)	There was a significant reduction in mortality rate of severely injured patients, but no significant difference in mortality rate of the entire patient population or resource utilization six months post-training
Bergman 2008 [53]	Tanzania	Trauma Team Training (TTT)	Nurses (13) Physicians (7)	Median post-course test scores (13/15) increased significantly compared to pre-course (9/15) knowledge of trauma management (p = 0.0004)
Mitchell, 2013 [54]	Tanzania	Essential Surgical Skills (ESS)	Medical students (60)	The study describes increased technical trauma surgical skills and confidence post-workshop
Carey, 2015 [55]	Tanzania	Surgical Management and Reconstructive Training course (SMART) for Orthopedic Trauma	Surgeons (34)	The SMART course resulted in 93.3% (554) successful flap procedures and aversion of 78.1% of potential amputations. Overall, the course cost USD 18,000–25,000
Lett, 2004 [56]	Uganda	Trauma Team Training (TTT)	Healthcare Professionals	All trainees noted gained practical skills and trauma teamwork and communication improved at hospitals. Course impact was noted to be reduced at health centers with limited staff and resources. The workshops cost USD 700–1000, not including initial USD 2000 for equipment
O’Hara, 2015 and O’Brien, 2018 [57, 58]	Uganda	Uganda Sustainable Trauma Orthopedic Program (USTOP)	Physicians, Surgeons, Nurses and Physiotherapists	USTOP was declared beneficial for all participants and made a positive impact on the surgical care of patients with orthopedic trauma injuries
Anderson, 2018 [59]	Uganda	“Surgical Techniques and Repairs in Trauma for the Low-resource Environment” (STaRTLE) and “Emergency Ward Management of Trauma” (EWMT)	Residents (STaRTLE = 8; EWMT = 15)	Average trauma management knowledge and understanding scores improved post-training by 19% and 23% for EWMT and STaRTLE participants, respectively. Additionally, there was high participant satisfaction across all aspects of the course (median 5/5 ratings)
Ullrich, 2020 [60, 61]	Uganda	Mulago Operative Trauma Resuscitation (M-OTR) Course / The Kampala Advanced Trauma Course (KATC)	Residents (28)	Although participants had significantly increased post-course versus pre-course test scores (p < 0.05), there was no significant increase in taught trauma operative management techniques
Edwards, 2011 [62]	Zambia	American Burn Association/Children's Burn Foundation (ABA/CBF) Team Training	Healthcare providers	There was no significant improvement in burn management parameters (surgical intervention, fluid resuscitation, skin grafting) from pre-course to post-course. Overall, any changes in patient management did not translate into improved patient outcomes
Tuggle, 2017 [63]	Zimbabwe	Advanced Trauma Care for Nurses (ATCN)	Nurses (64)	The study describes the first successful and sustainable implementation of ATCN/ATLS at a nursing college in Bulawayo

#### Participants and setting

The majority of studies reported on multidisciplinary courses (26/45, 57.8%), involving nurses, physicians, residents, medical students, medical/clinical officers, and other allied HCPs. Physicians and surgeons were the most common group of participants (27/45, 60.0%), followed by nurses (14/45, 31.1%), medical students (12/45, 26.7%), and residents (11/45, 24.4%). Other participants included in studies were unspecified HCPs (6/45, 13.3%), medical and/or clinical officers (5/45, 11.1%), rehabilitation therapists/physiotherapists (2/45, 4.4%), village health workers (1/45, 2.2%), emergency medical technicians (1/45, 2.2%), and/or midwives (1/45, 2.2%).

Included articles spanned 27 LMICs, with most of the studies (21/45, 46.7%) occurring in sub-Saharan Africa [38–63]. Nearly all of the studies were conducted in an urban setting (34/45, 75.6%), with only seven (15.6%) describing a rural-specific program [12, 13, 15, 31, 46, 49, 62] and four studies (8.9%) defining training in both urban and rural settings [17, 32, 41, 45].

#### Suitability and local engagement

Most studies engaged local stakeholders (43/45; 95.6%), in the form of co-creation or implementation of courses with local organizations (34/45; 75.6%); co-authorship including a member from the study setting (37/45; 82.2%); and/or study conducted by local stakeholders themselves (9/45; 20.0%). However, only 15/45 (33.3%) studies completed a formal needs assessment before implementing a trauma course. The needs assessments that were done examined factors such as availability of resources and personnel [14, 19, 24, 25, 29, 41, 44, 49, 60, 62, 63] local injury burden [24, 44, 60]; current training methods, gaps in training and future training requirements [14, 16, 19, 25, 29, 41, 46, 49, 56, 60, 63]; cost and suitability of the program [25, 41, 56, 62]; and optimal course delivery plans [16, 44, 60].

#### Courses breadth and competencies

Thirty-two different courses were described in retrieved studies, the majority of which focused on general surgical trauma training (34/45, 75.6%), with 14 of these courses being individualized for a specific setting in conjunction with a non-governmental organization (NGO) or high-income university. Of pre-established programs, PTC was the most commonly described course (7/45, 15.6%) [19, 26, 35, 36, 38, 40, 45], followed by Trauma Evaluation and Management (TEAM) (4/45, 8.9%) [23, 37, 48, 50], TTT (3/45, 6.7%) [52, 54, 56], and ATLS (3/45, 6.7%) [14, 30, 52].

Orthopedic trauma injury management was the focus of six (13.3%) studies [4, 18, 21, 22, 55, 57], while burn management was the focus of four, of which two reported on the implementation of the American Burn Association/Children's Burn Foundation (ABA/CBF) Team Training program [29, 62]. Only one study reported on trauma related to plastic surgery reconstruction [44].

#### Course effectiveness

Most studies found an improvement in trauma management skills and knowledge, regardless of course type (32/45, 71.1%). Most of the studies reported increase in participant skills and/or knowledge of trauma management based on pre-course versus post-course tests and surveys (28/45, 62.2%), while the remaining four studies evaluated knowledge through self-reflection surveys or interviews.

Twelve (26.7%) studies commented on changes to trauma management practices and patient outcomes post-course, of which seven had data on pre-course versus post-course outcomes [13, 19, 21, 29, 38, 46, 51, 52, 55, 58, 60, 62]. Only three studies objectively evaluated pre- to post-course changes in patient mortality and morbidity [19, 52, 62], of which two failed to find significant improvements [19, 62].

Cioè-Peña et al. [19] reported a significant increase in the use of FAST exams; however, other facets of trauma management, including critical skills and time to operating room, were not significantly altered by PTC training. Ullrich et al. [60] found an improvement from pre-course to post-course test scores, but actual trauma operative management techniques did not significantly improve.

Two studies implemented and examined ABA/CBF training on burn management competencies and patient outcomes [29, 62]. One study in India reported improved burn management skills, including decreased antibiotic use and earlier excision and grafting [29], while a similar study in Zambia did not report significant changes to burn management protocols or consequent patient outcomes [62].

Carey et al. [55] reported on the completion of 93.3% flaps with a 78.1% aversion rate of amputations, while Tajsic et al. [13] indicated a 70% primary healing rate of open fractures after orthopedic trauma courses. Participants of the Uganda Sustainable Trauma Orthopedic Program reported a subjective improvement for patients with orthopedic trauma [58]. Additionally, the orthopedic trauma PGME program in Malawi and Haiti demonstrated a significant improvement in patient outcomes post-training [21, 51].

ATLS training significantly decreased mortality of severely injured patients in Rwanda [52]. PTC training in El Salvador, and ABA/CBF training in Zambia did not result in improved patient mortality rates post-training [19, 62].

#### Sustainability

In total, 4/45 (8.9%) studies commented on course costs [14, 21, 55, 56], of which only one study examined cost-effectiveness of the course teachings versus traditional care [21]. The TTT workshops in Uganda were the most inexpensive, costing $700–1,000 USD/course, after an initial $2,000 USD was spent on equipment [56]. The Surgical Management and Reconstructive Training orthopedic trauma course in Tanzania was more expensive, with an annual cost of $18,000—25,000 USD [55]. A Mongolian ATLS study emphasized the benefit of training local staff as instructors, as annual course costs decreased from $68,038 USD to $10,709 USD when Mongolian trainers were used [14]. Carlson et al. [21] were the only study to report on the cost-effectiveness of their Orthopedic Trauma Care Specialist program, which resulted in cost savings of $134 USD per DALY per resident trained as an orthopedic trauma specialist surgeon.

In total, five studies followed participant outcomes for a period of at least six months, with only one study following up for greater than one year [45]. No studies described a recertification process. The two studies comparing retention of knowledge several months after course administration reported retained competencies in trauma knowledge and skills at nine months [49] and two years [45]. None of the studies examined long-term patient mortality and morbidity for a period greater than one year, nor compared results to pre-course parameters.

### Postgraduate medical education programs

**Fig. 2:**
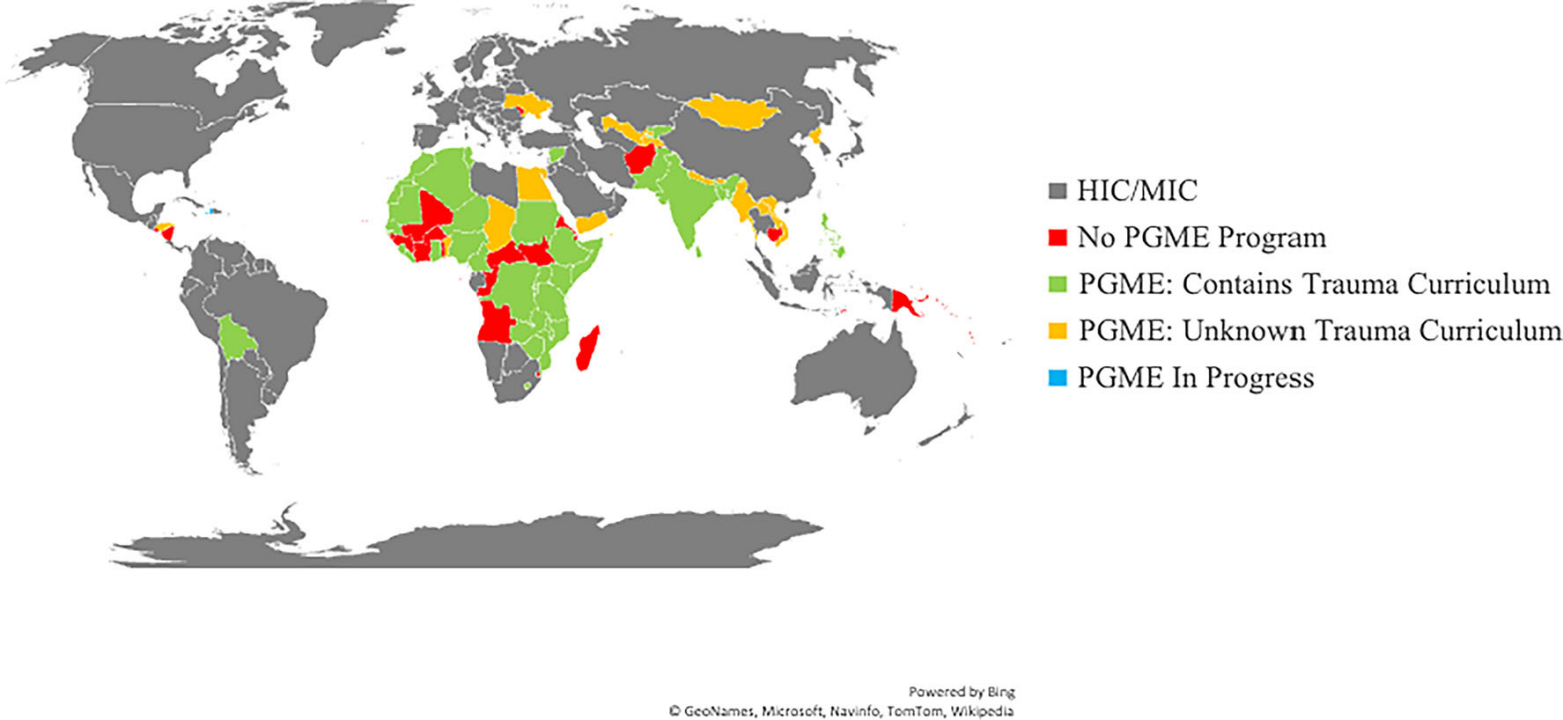
Distribution of surgical PGME programs and included trauma management competencies in low- and lower middle-income countries. Country income group determined by the World Bank Report, 2020. HIC, high-income country; MIC, middle-income country (including upper middle income); PGME, postgraduate medical education

In total, 51/79 (64.6%) of LMICs have a form of surgical PGME program, of which 34 (66.7%) define trauma skills as required curricular competencies (Fig. 2). These countries have formalized PGME programs in the form of residencies, fellowships, and Masters and/or Doctorates. Traumatology is most often a component of general surgery or orthopedic surgery PGME programs (Online Resource 3). Other PGME programs with required trauma competencies include neurosurgery, cardiothoracic surgery, pediatric surgery, maxillofacial surgery, urology, otorhinolaryngology, and plastic surgery. Most PGME programs are two to five years long, but time devoted to trauma training varies greatly between programs.

Several countries belong to an overarching professional society that regulates PGRME programs. In sub-Saharan Africa, this includes the College of Surgeons of East, Central, and Southern Africa (COSECSA) [8] and the West African College of Surgeons (WACS) [64]. The Arab Boards also regulate and accredit PGME programs in the Middle East, South Asia, and North Africa [65]. Although the American College of Surgeons (ACS) does not oversee PGME programs in LMICs, they do provide continuing medical education (CME) opportunities for trainees.

## Discussion

**Fig. 3:**
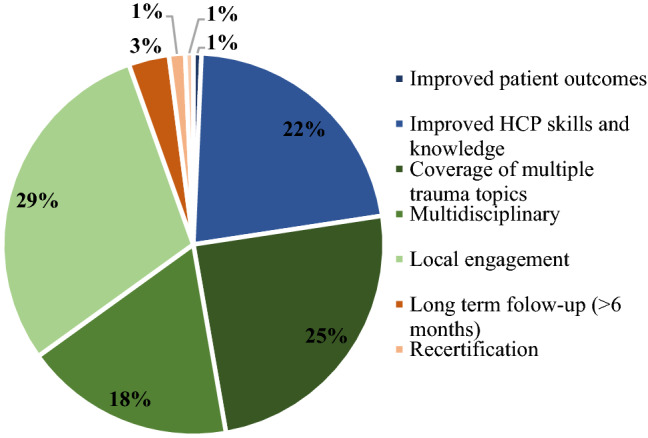
Number of courses demonstrating improvements in effectiveness, suitability, and sustainability of trauma training. Blue, effectiveness categories; green, suitability categories; and orange, sustainability categories

To our knowledge, this is the first review to provide a comprehensive overview of trauma training in LMICs, including both trauma courses and PGME programs. Overall, we discovered several attempts to implement various trauma training courses in LMICs, but there is little and conflicting evidence concerning the effectiveness, suitability, and sustainability of these courses long-term (Fig. 3).

### Inadequate needs assessments

Most of the studies attempted to engage locals when implementing trauma courses. However, only 15 studies conducted a formal needs assessment with their target populations. Successful trauma systems in LMICs include predevelopment needs assessments that identify existing resources and serve as a foundation for future developments [66]. Future studies should include formal needs assessments with stakeholders and may include methods such as the literature reviews, community surveys, focus groups, cross-sectional observations, chart reviews, or a combination [66]. However, although formal needs assessments were not conducted, several of the courses likely enshrined local engagement into their processes through longitudinal interactions with these populations and partnerships with LMIC colleagues.

### Lack of course standardization

Although many LMICs have PGME programs with trauma management competencies, the distribution, duration, and structure of these programs vary across countries and regions. These inconsistencies are further compounded by some countries belonging to multiple PGME regulatory organizations, resulting in different standards of accreditation for trainees in different specialties or hospitals across a given country. A cohesive and sustainable approach to trauma training, either regionally, nationally, or inter-regionally, has the opportunity to reduce costs and improve standardization and quality of education for HCPs involved in trauma care management.

### Indeterminate “best” trauma course

A reason for the lack of guidance on mandatory trauma courses may stem from uncertainty as to the most beneficial trauma course for LMICs. Contributing to the uncertainty in course superiority is the fact that studies have failed to demonstrate equal or improved care by alternative courses in LMICs compared to ATLS, the current gold standard for trauma training in HICs [67]. The fact that the two non-ATLS courses reporting on changes in patient mortality revealed no significant difference in pre-course and post-course patient outcomes creates a question to the utility and validity of these courses in trauma education [19, 62].

ATLS was the only program to demonstrate concrete mortality benefits to injured patients; however, ATLS lacks a multidisciplinary approach that reflects the reality in LMICs. Rural Trauma Team Development Course (RTTDC) is also offered by the ACS and provides multidisciplinary trauma training and emphasizes teamwork and crisis resource management and has shown success in improving outcomes in rural settings [68]. Only one study looked at RTTDC implementation and did not link course participation to patient outcomes. However, a hybrid model of RTTDC and ATLS combining the multidisciplinary and resource management components of RTTDC with the high standards and quality of training of ATLS may be beneficial in these regions.

The lack of cost-effectiveness data of courses further adds to the ambiguity surrounding their appropriateness in an LMIC setting. Several previous studies have claimed the expensive nature of ATLS, along with reliance on high-income equipment, and make the course unsuitable for use in LMICs [52]. However, we have found a lack of evidence to support the cost-effectiveness of other trauma training courses in LMICs. Evidently, a robust health economics evaluation of the different programs, models of training, resource utilization, and patient outcomes are needed before conclusive statements on course cost-effectiveness can be made.

Overall, limitations in course evaluation with respect to cost-effectiveness and clinical outcomes can be partially attributed to the insufficient follow-up time of the studies. An essential component to CME is the process of recertification, something enshrined in the ATLS model and recommended in the WHO’s Guidelines for trauma quality improvement programs [69]. None of the included studies mentioned a recertification process for participants after taking a stand-alone trauma course. This brings up an issue of sustainability and ethical responsibility to ensure trainees have opportunities to refresh their knowledge and skills, representing an area of needed improvement in trauma training in these regions. There is a substantial lack of funding for global surgery initiatives, and many of these courses are paid for through donations. A stronger global surgery funding network would bolster the scientific rigor of this subject and allow for improved outcome measurements, including longer follow-up periods.

### Limitations

These findings should be interpreted with respect to limitations of this study. This review was only able to capture information on trauma programs published online. Furthermore, the search strategy was conducted in English and therefore may have missed articles or websites in different languages. Consequently, PGME programs may exist that were not identified. Additionally, available websites for PGME programs may not be up to date or reflect actual functioning of the programs.

NGOs such as Médecins Sans Frontières and the International Committee of the Red Cross train HCPs during their deployments in trauma care. These trainings are not typically published as research articles and traditionally rely on HCPs from HICs and therefore were outside of the scope of this review. However, these organizations are increasingly providing multidisciplinary trauma training to local practitioners and are important collaborators and funders of trauma training in LMICs.

Furthermore, we excluded laypeople trained in trauma care. Laypeople play an important role in trauma management in LMICs, especially in rural and pre-hospital settings [70]. However, in an attempt to succinctly define parameters of this study, we chose to focus exclusively on trained HCPs providing definitive trauma care. This narrowed the scope of our results, providing a reductionist picture of the trauma care as a whole in LMICs.

## Conclusion

**Fig. 4:**
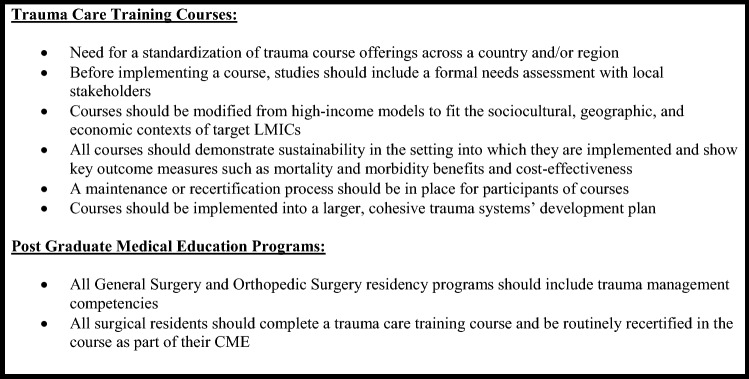
Summary of recommendations

This scoping review is the first known study to provide a comprehensive examination of trauma training opportunities, including PGME programs in LMICs. Overall, this study has illuminated the notable lack of standardization and evidence-based implementation of trauma training in these regions. It highlights the fragmentation in trauma training in many LMICs when well-intentioned players, often from HICs, introduce multiple courses without needs-informed engagement from local stakeholders. Although several different trauma courses have been executed in LMICs, studies have failed to prove long-term mortality, morbidity, and/or economic benefits. Trauma training programs should be required to demonstrate these outcomes and establish a standardized and longitudinal process for HCP trauma certification in a given country or region. A comprehensive and effective trauma education program should include short courses like ATLS that are contextualized to the setting, incorporated into CME, and demonstrate tangible benefits to patients and the healthcare system (Fig. 4).

## Supplementary Information

Below is the link to the electronic supplementary material.

Supplementary file1 (PDF 262 kb)

Supplementary file2 (PDF 190 kb)

Supplementary file3 (PDF 239 kb)
