# Supplementary material for: Trauma Training Courses and Programs in Low- and Lower Middle-Income Countries: A Scoping Review
Source: World J Surg. 2021 Sep 5;45(12):3543–57. doi: 10.1007/s00268-021-06283-1 (PMC8572832; doi:10.1007/s00268-021-06283-1)
Supplement: Supplementary file 2 — Supplementary file2 (PDF 190 kb) [file 268_2021_6283_MOESM2_ESM.pdf]

Title: Trauma training courses and programs in low- and lower- middle income countries: A scoping review.

Journal: World Journal of Surgery

Authors:

Rachel J. Livergant, MBT[1]; Selina Demetrick, BSc[1]; Xenia Cravetchi, MSc[1]; Janice Y. Kung, MLIS[2], Emilie Joos, FRCSC/FACS[3]; Harvey G. Hawes, FRCSC[3]; Abdullah Saleh, FRCSC[1*]

Affiliations:

[1]Office of Global Surgery, Department of Surgery, University of Alberta, Edmonton, Canada

[2]John W. Scott Health Sciences Library, University of Alberta, Edmonton, Canada

[3]Division of General Surgery, Trauma and Acute Care Surgery, Vancouver General Hospital, University of British Columbia, Vancouver, British Columbia

Addresses:

[1]2D2.23 Walter Mackenzie Health Sciences Centre, 8440 - 112 Ave NW, Edmonton, Alberta, T6G 2B7, Canada

[2]2K3.28 Walter C. Mackenzie Health Sciences Centre, 8440 - 112 Ave NW, Edmonton, Alberta, T6G 2R7, Canada

[3]Jim Pattison Pavilion, 899 W 12th Ave, Vancouver, British Columbia V5Z 1M9, Canada

*Corresponding author: Dr. Abdullah Saleh; Department of Surgery, Office of Global Surgery, University of Alberta, 2D2.23 Walter Mackenzie Health Sciences Centre, 8440 - 112 Ave NW, Edmonton, Alberta, T6G 2B7, Canada; Tel: +403-973-7974; Fax: 780-407-2004; Email: aasaleh@ualberta.ca

Online Resource 2. Search strategies from electronic database searches.

**Database**	**Search Strategy**
**MEDLINE**<br><br>Ovid MEDLINE(R) ALL 1946 to August 21, 2020	1. (Advanced Trauma Life Support or ATLS).mp.<br>2. American Burn Association.mp.<br>3. Children* Burn Foundation.mp.<br>4. Primary Trauma Care.mp.<br>5. Trauma Team Training.mp.<br>6. National Trauma Management Course.mp.<br>7. Essential Surgical Skills.mp.<br>8. exp *Trauma Centers/og [Organization & Administration]<br>9. ((acute care or trauma or critical care or triage or emergicent* or casualty department*) adj5 (train* or program* or course* or education* or simulation* or learn* or teach* or set* up)).mp.<br>10. ((emergenc* or ED) adj1 (room* or accident or ward or wards or unit or units or department* or physician* or doctor* or nurs* or treatment* or visit* or service*) adj5 (train* or program* or course* or education* or simulation* or learn* or teach* or set* up)).mp.<br>11. Emergency Treatment/ or Emergency Medicine/ or emergency medical services/ or emergency service, hospital/ or Trauma Centers/ or Triage/ or exp Evidence-Based Emergency Medicine/ or exp Emergency Nursing/ or Emergencies/<br>12. ((train* or program* or course* or education* or simulation* or learn* or teach* or set* up) adj5 (physician* or doctor* or clinician* or medical officer* or nurs* or practitioner* or worker*)).ti,ab,kf.<br>13. 11 and 12<br>14. 1 or 2 or 3 or 4 or 5 or 6 or 7 or 8 or 9 or 10 or 13<br>15. afghanistan/ or algeria/ or angola/ or bangladesh/ or benin/ or bhutan/ or bolivia/ or burkina faso/ or burundi/ or cabo verde/ or cambodia/ or cameroon/ or central african republic/ or chad/ or comoros/ or congo/ or cote d'ivoire/ or "democratic people's republic of korea"/ or "democratic republic of the congo"/ or djibouti/ or egypt/ or el salvador/ or eritrea/ or eswatini/ or ethiopia/ or gambia/ or ghana/ or guinea-bissau/ or guinea/ or haiti/ or honduras/ or india/ or kenya/ or kiribati/ or kyrgyzstan/ or laos/ or lesotho/ or liberia/ or madagascar/ or malawi/ or mali/ or mauritania/ or melanesia/ or micronesia/ or moldova/ or mongolia/ or morocco/ or mozambique/ or myanmar/ or nepal/ or nicaragua/ or nigeria/ or niger/ or pakistan/ or papua new guinea/ or philippines/ or rwanda/ or "sao tome and principe"/ or senegal/ or sierra leone/ or somalia/ or south sudan/ or sri lanka/ or sudan/ or syria/ or tajikistan/ or tanzania/ or timor-leste/ or togo/ or tunisia/ or uganda/ or ukraine/ or uzbekistan/ or vanuatu/ or vietnam/ or yemen/ or zambia/ or zimbabwe/<br>16. (Afghanistan or Afghani or Afghan or (Africa* not America*) or Algeria* or Angola* or Bangladesh* or Benin or Beninese or Bhutan or Bolivia* or Burkina Faso or Burundi* or Cape Verde or Cabo Verde or Cambodia* or Cameroon* or Central African Republic or Chad or Chadian or Tchad or Comoros or Congo or Congolese or Cote d'Ivoire or Ivorian or Djibouti or Egypt or Egyptian* or El Salvador or Salvadoran* or Eritrea* or Eswatini or Swaziland or Swazi or Ethiopia* or Gambia or Gambian or Ghana or (Guinea not pig) or Guinea-Bissau or Haiti or Haitian or Hondura* or India or (Indian not American) or Kenya* or Kiribati or "Democratic People* Republic of Korea" or North Korea* or DPRK or Kyrgyz* or (Lao and People* Democratic Republic) or Lao PDR or Laos or Laotian or Lesotho or Mosotho or Basotho or Liberia* or Madagascar or Malagasy or Malawi* or Mali or Malian or Mauritania* or Melanesia* or Micronesia* or Moldova* or Mongolia* or Morocco or Moroccan or Mozambique or Mozambican or Myanmar or Burmese or Myanmarese or Nepal or Nepalese or Nicaragua* or Niger or Nigerien or Nigeria or Nigerian or Pakistan* or Papua New Guinea* or Philippines or Filipino* or Rwanda* or "Sao Tome and Principe" or Sao Tomean or Santomean or San Tomean or Senegal* or Sierra Leone* or Solomon Island* or

	Somalia* or South* Asia* or South Sudan or Sri Lanka* or Sudan or Sudanese or Syria or Syrian or Tajikistan or Tajik or Tadzhik or Tanzania* or Timor Leste or Timorese or Togo or Togolese or Tunisia* or Uganda* or Ukraine or Ukrainian or Uzbekistan* or Uzbeki or Vanuatu or Vietnam* or West Bank or Gaza or Yemen* or Zambia* or Zimbabwe*).ti,ab,cp.<br><br>17. (("low* and middle income" or low middle income or low income) adj1 (setting* or nation* or countr* or region*)).ti,ab,kf.<br>18. LMIC.ti,ab,kf.<br>19. ((low resource or underdeveloped or under developed or developing) adj1 (setting* or nation* or countr* or region*)).ti,ab,kf.<br>20. or/15-19<br>21. 14 and 20<br>22. limit 21 to yr="2000 -Current"
**Embase**<br><br>Ovid Embase 1974 to 2020 August 21	1. (Advanced Trauma Life Support or ATLS).mp.<br>2. exp advanced trauma life support care/<br>3. American Burn Association.mp.<br>4. Children* Burn Foundation.mp.<br>5. Primary Trauma Care.mp.<br>6. Trauma Team Training.mp.<br>7. National Trauma Management Course.mp.<br>8. Essential Surgical Skills.mp.<br>9. ((acute care or trauma or critical care or triage or emergicent* or casualty department*) adj5 (train* or program* or course* or education* or simulation* or learn* or teach* or set* up)).mp.<br>10. ((emergenc* or ED) adj1 (room* or accident or ward or wards or unit or units or department* or physician* or doctor* or nurs* or treatment* or visit* or service*) adj5 (train* or program* or course* or education* or simulation* or learn* or teach* or set* up)).mp.<br>11. emergency treatment/ or emergency medicine/ or exp emergency health service/ or evidence based emergency medicine/ or emergency nursing/ or exp emergency care/ or emergency ward/ or emergency/<br>12. ((train* or program* or course* or education* or simulation* or learn* or teach* or set* up) adj5 (physician* or doctor* or clinician* or medical officer* or nurs* or practitioner* or worker*)).ti,ab,kw.<br>13. 11 and 12<br>14. 1 or 2 or 3 or 4 or 5 or 6 or 7 or 8 or 9 or 10 or 13<br>15. afghanistan/ or algeria/ or angola/ or bangladesh/ or benin/ or bhutan/ or bolivia/ or burkina faso/ or burundi/ or cambodia/ or cameroon/ or cape verde/ or central african republic/ or chad/ or comoros/ or congo/ or cote d'ivoire/ or democratic republic congo/ or djibouti/ or egypt/ or el salvador/ or eritrea/ or eswatini/ or ethiopia/ or exp "Federated States of Micronesia"/ or gambia/ or ghana/ or guinea-bissau/ or guinea/ or haiti/ or honduras/ or exp india/ or kenya/ or kiribati/ or kyrgyzstan/ or laos/ or lesotho/ or liberia/ or madagascar/ or malawi/ or mali/ or mauritania/ or moldova/ or mongolia/ or morocco/ or mozambique/ or myanmar/ or nepal/ or nicaragua/ or niger/ or nigeria/ or north korea/ or exp pakistan/ or papua new guinea/ or philippines/ or rwanda/ or "sao tome and principe"/ or senegal/ or sierra leone/ or solomon islands/ or exp somalia/ or south sudan/ or sri lanka/ or sudan/ or syrian arab republic/ or tajikistan/ or tanzania/ or timor-leste/ or togo/ or tunisia/ or uganda/ or ukraine/ or uzbekistan/ or vanuatu/ or viet nam/ or yemen/ or zambia/ or zimbabwe/<br>16. (Afghanistan or Afghani or Afghan or (Africa* not America*) or Algeria* or Angola* or Bangladesh* or Benin or Beninese or Bhutan or Bolivia* or Burkina Faso or Burundi* or Cape Verde or Cabo Verde or Cambodia* or Cameroon* or Central African Republic or Chad or Chadian or Tchad or Comoros or Congo or Congolese or Cote d'Ivoire or Ivorian or Djibouti or Egypt or Egyptian* or El Salvador or Salvadoran* or Eritrea* or Eswatini or Swaziland or Swazi or Ethiopia* or Gambia or Gambian or Ghana or (Guinea not pig) or Guinea-Bissau or Haiti or Haitian or Hondura* or India or (Indian not American) or Kenya*

	or Kiribati or "Democratic People* Republic of Korea" or North Korea* or DPRK or Kyrgyz* or (Lao and People* Democratic Republic) or Lao PDR or Laos or Laotian or Lesotho or Mosotho or Basotho or Liberia* or Madagascar or Malagasy or Malawi* or Mali or Malian or Mauritania* or Melanesia* or Micronesia* or Moldova* or Mongolia* or Morocco or Moroccan or Mozambique or Mozambican or Myanmar or Burmese or Myanmarese or Nepal or Nepalese or Nicaragua* or Niger or Nigerien or Nigeria or Nigerian or Pakistan* or Papua New Guinea* or Philippines or Filipino* or Rwanda* or "Sao Tome and Principe" or Sao Tomean or Santomean or San Tomean or Senegal* or Sierra Leone* or Solomon Island* or Somalia* or South* Asia* or South Sudan or Sri Lanka* or Sudan or Sudanese or Syria or Syrian or Tajikistan or Tajik or Tadzhik or Tanzania* or Timor Leste or Timorese or Togo or Togolese or Tunisia* or Uganda* or Ukraine or Ukrainian or Uzbekistan* or Uzbeki or Vanuatu or Vietnam* or West Bank or Gaza or Yemen* or Zambia* or Zimbabwe*).ti,ab,cp.<br><br>17. (("low* and middle income" or low middle income or low income) adj1 (setting* or nation* or countr* or region*)).ti,ab,kw.<br>18. LMIC.ti,ab,kw.<br>19. ((low resource or underdeveloped or under developed or developing) adj1 (setting* or nation* or countr* or region*)).ti,ab,kw.<br>20. or/15-19<br>21. 14 and 20<br>22. limit 21 to yr="2000 -Current"
**Global Health**<br><br>Ovid Global Health 1973 to 2020 Week 33	1. (Advanced Trauma Life Support or ATLS).mp.<br>2. American Burn Association.mp.<br>3. Children* Burn Foundation.mp.<br>4. Primary Trauma Care.mp.<br>5. Trauma Team Training.mp.<br>6. National Trauma Management Course.mp.<br>7. Essential Surgical Skills.mp.<br>8. ((acute care or trauma or critical care or triage or emergicent* or casualty department*) adj5 (train* or program* or course* or education* or simulation* or learn* or teach* or set* up)).mp.<br>9. ((emergenc* or ED) adj1 (room* or accident or ward or wards or unit or units or department* or physician* or doctor* or nurs* or treatment* or visit* or service*) adj5 (train* or program* or course* or education* or simulation* or learn* or teach* or set* up)).mp.<br>10. 1 or 2 or 3 or 4 or 5 or 6 or 7 or 8 or 9<br>11. (Afghanistan or Afghani or Afghan or (Africa* not America*) or Algeria* or Angola* or Bangladesh* or Benin or Beninese or Bhutan or Bolivia* or Burkina Faso or Burundi* or Cape Verde or Cabo Verde or Cambodia* or Cameroon* or Central African Republic or Chad or Chadian or Tchad or Comoros or Congo or Congolese or Cote d'Ivoire or Ivorian or Djibouti or Egypt or Egyptian* or El Salvador or Salvadoran* or Eritrea* or Eswatini or Swaziland or Swazi or Ethiopia* or Gambia or Gambian or Ghana or (Guinea not pig) or Guinea-Bissau or Haiti or Haitian or Hondura* or India or (Indian not American) or Kenya* or Kiribati or "Democratic People* Republic of Korea" or North Korea* or DPRK or Kyrgyz* or (Lao and People* Democratic Republic) or Lao PDR or Laos or Laotian or Lesotho or Mosotho or Basotho or Liberia* or Madagascar or Malagasy or Malawi* or Mali or Malian or Mauritania* or Melanesia* or Micronesia* or Moldova* or Mongolia* or Morocco or Moroccan or Mozambique or Mozambican or Myanmar or Burmese or Myanmarese or Nepal or Nepalese or Nicaragua* or Niger or Nigerien or Nigeria or Nigerian or Pakistan* or Papua New Guinea* or Philippines or Filipino* or Rwanda* or "Sao Tome and Principe" or Sao Tomean or Santomean or San Tomean or Senegal* or Sierra Leone* or Solomon Island* or Somalia* or South* Asia* or South Sudan or Sri Lanka* or Sudan or Sudanese or Syria or Syrian or Tajikistan or Tajik or Tadzhik or Tanzania* or Timor Leste or Timorese or Togo or Togolese or Tunisia* or Uganda* or Ukraine or Ukrainian or Uzbekistan* or Uzbeki or Vanuatu or Vietnam* or West Bank or Gaza or Yemen* or Zambia* or Zimbabwe*).mp.

	12. (("low* and middle income" or low middle income or low income) adj1 (setting* or nation* or countr* or region*)).mp.<br>13. LMIC.ti,ab.<br>14. ((low resource or underdeveloped or under developed or developing) adj1 (setting* or nation* or countr* or region*)).mp.<br>15. or/11-14<br>16. 10 and 15<br>17. limit 16 to yr="2000 -Current"
**Cochrane Library**	#1 "Advanced Trauma Life Support" or ATLS<br>#2 "American Burn Association"<br>#3 "Children* Burn Foundation"<br>#4 "Primary Trauma Care"<br>#5 "Trauma Team Training"<br>#6 "National Trauma Management Course"<br>#7 "Essential Surgical Skills"<br>#8 [mh "Trauma Centers"/og]<br>#9 ("acute care" or trauma or "critical care" or triage or emergicent* or "casualty department*") NEAR/5 (train* or program* or course* or education* or simulation* or learn* or teach* or "set* up")<br>#10 (emergenc* or ED) NEXT (room* or accident or ward or wards or unit or units or department* or physician* or doctor* or nurs* or treatment* or visit* or service*)<br>#11 (room* or accident or ward or wards or unit or units or department* or physician* or doctor* or nurs* or treatment* or visit* or service*) NEAR/5 (train* or program* or course* or education* or simulation* or learn* or teach* or "set* up")<br>#12 #10 AND #11<br>#13 {OR #1-#9}<br>#14 #12 OR #13<br>#15 (Afghanistan or Afghani or Afghan or (Africa* not America*) or Algeria* or Angola* or Bangladesh* or Benin or Beninese or Bhutan or Bolivia* or Burkina Faso or Burundi* or Cape Verde or Cabo Verde or Cambodia* or Cameroon* or "Central African Republic" or Chad or Chadian or Tchad or Comoros or Congo or Congolese or Cote d'Ivoire or Ivorian or Djibouti or Egypt or Egyptian* or El Salvador or Salvadoran* or Eritrea* or Eswatini or Swaziland or Swazi or Ethiopia* or Gambia or Gambian or Ghana or (Guinea not pig) or Guinea-Bissau or Haiti or Haitian or Hondura* or India or (Indian not American) or Kenya* or Kiribati or "Democratic People* Republic of Korea" or North Korea* or DPRK or Kyrgyz* or "Lao and People* Democratic Republic" or Lao PDR or Laos or Laotian or Lesotho or Mosotho or Basotho or Liberia* or Madagascar or Malagasy or Malawi* or Mali or Malian or Mauritania* or Melanesia* or Micronesia* or Moldova* or Mongolia* or Morocco or Moroccan or Mozambique or Mozambican or Myanmar or Burmese or Myanmarese or Nepal or Nepalese or Nicaragua* or Niger or Nigerien or Nigeria or Nigerian or Pakistan* or Papua New Guinea* or Philippines or Filipino* or Rwanda* or "Sao Tome and Principe" or Sao Tomean or Santomean or San Tomean or Senegal* or Sierra Leone* or Solomon Island* or Somalia* or "South* Asia*" or South Sudan or Sri Lanka* or Sudan or Sudanese or Syria or Syrian or Tajikistan or Tajik or Tadzhik or Tanzania* or Timor Leste or Timorese or Togo or Togolese or Tunisia* or Uganda* or Ukraine or Ukrainian or Uzbekistan* or Uzbeki or Vanuatu or Vietnam* or "West Bank" or Gaza or Yemen* or Zambia* or Zimbabwe*):ti,ab<br>#16 ("low* and middle income" or "low middle income" or "low income") NEXT (setting* or nation* or countr* or region*)<br>#17 LMIC:ti,ab<br>#18 ("low resource" or underdeveloped or "under developed" or developing) NEXT (setting* or nation* or countr* or region*)<br>#19 {OR #15-#18}

	#20 #14 AND #19<br><br>Custom Date Range: 2000 - current
**ProQuest Dissertations & Theses Global**	noft("Advanced Trauma Life Support" or ATLS or "American Burn Association" or "Children* Burn Foundation" or "Primary Trauma Care" or "Trauma Team Training" or "National Trauma Management Course" or "Essential Surgical Skills" or (("acute care") NEAR/5 (train* or program* or course* or education* or simulation* or learn* or teach* or "set* up")) or ((trauma) NEAR/5 (train* or program* or course* or education* or simulation* or learn* or teach* or "set* up")) or (("critical care") NEAR/5 (train* or program* or course* or education* or simulation* or learn* or teach* or "set* up")) or ((triage) NEAR/5 (train* or program* or course* or education* or simulation* or learn* or teach* or "set* up")) or (("casualty department*") NEAR/5 (train* or program* or course* or education* or simulation* or learn* or teach* or "set* up")) or ((emergen*) NEAR/5 (train* or program* or course* or education* or simulation* or learn* or teach* or "set* up")) or ((trauma) NEAR/5 (train* or program* or course* or education* or simulation* or learn* or teach* or "set* up")) or ((ED) NEAR/5 (train* or program* or course* or education* or simulation* or learn* or teach* or "set* up")) or ((trauma) NEAR/5 (train* or program* or course* or education* or simulation* or learn* or teach* or "set* up"))) AND noft((Afghanistan or Afghani or Afghan or (Africa* not America*) or Algeria* or Angola* or Bangladesh* or Benin or Beninese or Bhutan or Bolivia* or Burkina Faso or Burundi* or Cape Verde or Cabo Verde or Cambodia* or Cameroon* or "Central African Republic" or Chad or Chadian or Tchad or Comoros or Congo or Congolese or Cote d'Ivoire or Ivorian or Djibouti or Egypt or Egyptian* or El Salvador or Salvadoran* or Eritrea* or Eswatini or Swaziland or Swazi or Ethiopia* or Gambia or Gambian or Ghana or (Guinea not pig) or Guinea-Bissau or Haiti or Haitian or Hondura* or India or (Indian not American) or Kenya* or Kiribati or "Democratic People* Republic of Korea" or "North Korea*" or DPRK or Kyrgyz* or "Lao and People* Democratic Republic" or Lao PDR or Laos or Laotian or Lesotho or Mosotho or Basotho or Liberia* or Madagascar or Malagasy or Malawi* or Mali or Malian or Mauritania* or Melanesia* or Micronesia* or Moldova* or Mongolia* or Morocco or Moroccan or Mozambique or Mozambican or Myanmar or Burmese or Myanmarese or Nepal or Nepalese or Nicaragua* or Niger or Nigerien or Nigeria or Nigerian or Pakistan* or Papua New Guinea* or Philippines or Filipino* or Rwanda* or "Sao Tome and Principe" or Sao Tomean or Santomean or San Tomean or Senegal* or Sierra Leone* or Solomon Island* or Somalia* or South* Asia* or South Sudan or Sri Lanka* or Sudan or Sudanese or Syria or Syrian or Tajikistan or Tajik or Tadzhik or Tanzania* or Timor Leste or Timorese or Togo or Togolese or Tunisia* or Uganda* or Ukraine or Ukrainian or Uzbekistan* or Uzbeki or Vanuatu or Vietnam* or "West Bank" or Gaza or Yemen* or Zambia* or Zimbabwe*) or (("low* and middle income" or "low middle income" or "low income") NEXT (setting* or nation* or countr* or region*)) or LMIC or (("low resource" or underdeveloped or "under developed" or developing) NEXT (setting* or nation* or countr* or region*)))<br><br>Date Range: 2000 - current
**Google Scholar**	("Advanced Trauma Life Support" or acute care or emergency service or trauma care) AND (training or teaching) AND (low middle income countries or LMIC or lower income countries or low resource settings)
