# Supplementary material for: Trauma Training Courses and Programs in Low- and Lower Middle-Income Countries: A Scoping Review
Source: World J Surg. 2021 Sep 5;45(12):3543–57. doi: 10.1007/s00268-021-06283-1 (PMC8572832; doi:10.1007/s00268-021-06283-1)
Supplement: Supplementary file 3 — Supplementary file3 (PDF 239 kb) [file 268_2021_6283_MOESM3_ESM.pdf]

Title: Trauma training courses and programs in low- and lower- middle income countries: A scoping review.

Journal: World Journal of Surgery

Authors:

Rachel J. Livergant, MBT[1]; Selina Demetrick, BSc[1]; Xenia Cravetchi, MSc[1]; Janice Y. Kung, MLIS[2], Emilie Joos, FRCSC/FACS[3]; Harvey G. Hawes, FRCSC[3]; Abdullah Saleh, FRCSC[1*]

Affiliations:

[1]Office of Global Surgery, Department of Surgery, University of Alberta, Edmonton, Canada

[2]John W. Scott Health Sciences Library, University of Alberta, Edmonton, Canada

[3]Division of General Surgery, Trauma and Acute Care Surgery, Vancouver General Hospital, University of British Columbia, Vancouver, British Columbia

Addresses:

[1]2D2.23 Walter Mackenzie Health Sciences Centre, 8440 - 112 Ave NW, Edmonton, Alberta, T6G 2B7, Canada

[2]2K3.28 Walter C. Mackenzie Health Sciences Centre, 8440 - 112 Ave NW, Edmonton, Alberta, T6G 2R7, Canada

[3]Jim Pattison Pavilion, 899 W 12th Ave, Vancouver, British Columbia V5Z 1M9, Canada

*Corresponding author: Dr. Abdullah Saleh; Department of Surgery, Office of Global Surgery, University of Alberta, 2D2.23 Walter Mackenzie Health Sciences Centre, 8440 - 112 Ave NW, Edmonton, Alberta, T6G 2B7, Canada; Tel: +403-973-7974; Fax: 780-407-2004; Email: aasaleh@ualberta.ca.

Online Resource 3. Description of postgraduate medical education programs available in low- and lower-middle income countries.

Country	PGME Program?	Surgical Specialties	Trauma Competency Description	Duration	International Organization Affiliation
Afghanistan	None found.	NA	NA	NA	
Algeria	YES	General Surgery	Required competencies in specialty-specific trauma scenarios.	5 years	Arab Boards
Angola	None found.	NA	NA	NA	
Bangladesh	YES	General Surgery	Emergency surgery and burn management	5 years	ACS
Benin	YES	Cranio-Facial Surgery<br>General Surgery<br>Otorhinolaryngology	Not reported.	4 years	
Bhutan	YES	General Surgery<br>Orthopedic Surgery<br>Otorhinolaryngology	Not reported.	Not reported.	
Bolivia	YES	General Surgery<br>Pediatric Surgery<br>Otorhinolaryngology<br>Traumatology and Orthopedics<br>Urology	Required competencies in specialty-specific trauma scenarios.	3 - 4 years	ACS
Burkina Faso	None found.	NA	NA	NA	
Burundi	YES	General Surgery<br>Orthopedics<br>Otorhinolaryngology	Trauma competencies and placements required for all specialties (1 - 3 years) and required Critical Care or Trauma course	2 - 5 years	COSECSA
Cabo Verde	None found.	NA	NA	NA	
Cambodia	None found.	NA	NA	NA	
Cameroon	YES	General Surgery<br>Surgery	6 months trauma training for all programs with additional training in speciality-specific trauma scenarios.	2 - 5 years	WACS

Central African Republic	None found.	NA	NA	NA	
Chad	YES	General Surgery	Not reported.	Not reported.	
Comoros	None found.	NA	NA	NA	
Congo, Dem. Rep.	YES	Surgery	Two months training in emergency medicine and trauma	2 years	COSECSA
Congo, Rep.	None found.	NA	NA	NA	
Côte d'Ivoire	None found.	NA	NA	NA	
Djibouti	None found.	NA	NA	NA	
Egypt, Arab Rep.	YES	Cardiothoracic Surgery<br>General Surgery<br>Neurosurgery<br>Otolaryngology<br>Urology	Not reported.	5 years	Arab Boards
El Salvador	None found.	NA	NA	NA	
Eritrea	None found.	NA	NA	NA	
Eswatini	None found.	NA	NA	NA	
Ethiopia	YES	Cardiothoracic Surgery<br>General Surgery<br>Neurosurgery<br>Orthopedic Surgery<br>Otorhinolaryngology<br>Pediatric Surgery<br>Pediatric Orthopedic Surgery<br>Plastic Surgery<br>Surgery<br>Urology	Trauma competencies and placements required for all specialties (1 - 3 years) and required Critical Care or Trauma course	2 - 5 years	COSECSA
Gambia, The	YES	General Surgery<br>Surgery	6 months trauma training for all programs with additional training in speciality-specific trauma scenarios.	3 - 5 years	WACS

Ghana	YES	Cardiothoracic Surgery<br>General Surgery<br>Neurosurgery<br>Orthopedics and Traumatology<br>Pediatric Surgery<br>Plastic Surgery<br>Surgery<br>Urology	6 months trauma training for all programs with additional training in speciality-specific trauma scenarios.	3 - 8 years	WACS
Guinea	None found.	NA	NA	NA	
Guinea-Bissau	None found.	NA	NA	NA	
Haiti	In Progress	General Surgery<br>Orthopedic Surgery	Not reported.	Not reported.	
Honduras	YES	General Surgery<br>Neurosurgery<br>Plastic Surgery	Not reported.	Not reported.	
India	YES	General Surgery<br>Orthopedics<br>Otorhinolaryngology<br>Trauma and Surgery	Required competencies in specialty-specific trauma scenarios. Requirement of completion of ATLS workshop. Optional course in Advanced Burn Life Support.	2 - 3 years	ACS / Arab Boards
Kenya	YES	Cardiothoracic Surgery<br>General Surgery<br>Neurosurgery<br>Otorhinolaryngology<br>Pediatric Surgery<br>Plastic Surgery<br>Surgery<br>Urology	Trauma competencies and placements required for all specialties (1 - 3 years) and required Critical Care or Trauma course	2 - 5 years	COSECSA
Kiribati	None found.	NA	NA	NA	
Korea, Dem. People's Rep.	YES	Not reported.	Not reported.	Not reported.	
Kyrgyz Republic	YES	Cardiovascular Surgery<br>Emergency Medical Aid Surgery<br>General Surgery<br>Neurosurgery<br>Otolaryngology<br>Pediatric Surgery<br>Plastic Surgery<br>Traumatology and Orthopedics<br>Urology	Required competencies in specialty-specific trauma scenarios.	Minimum 2 years	
Lao PDR	YES	Surgery	Not reported.	2 - 3 years	

Lesotho	YES	General Surgery<br>Surgery	Trauma competencies and placements required for all specialties (1 - 3 years) and required Critical Care or Trauma course	2 - 5 years	COSECSA
Liberia	YES	Surgery	6 months trauma training.	3 years	WACS
Madagascar	None found.	NA	NA	NA	
Malawi	YES	General Surgery<br>Neurosurgery<br>Otorhinolaryngology<br>Orthopedic Surgery<br>Pediatric Surgery<br>Plastic Surgery<br>Surgery<br>Urology	Trauma competencies and placements required for all specialties (1 - 3 years) and required Critical Care or Trauma course	2 - 5 years	COSECSA
Mali	None found.	NA	NA	NA	
Mauritania	YES	General Surgery<br>Neurosurgery<br>Otorhinolaryngology<br>Urology	Trauma competencies in neurotrauma, maxillo-facial trauma, urological trauma and abdominal trauma	1 - 5 years	
Micronesia, Fed. Sts.	None found.	NA	NA	NA	
Moldova	None found.	NA	NA	NA	
Mongolia	YES	Not reported.	Not reported.	Not reported.	
Morocco	YES	Emergency Surgery<br>General Surgery<br>Orthopedics and Traumatology	Orthopedics; Acute General Surgery	5 years	
Mozambique	YES	General Surgery<br>Orthopedic Surgery<br>Pediatric Surgery<br>Surgery<br>Urology	Trauma competencies and placements required for all specialties (1 - 3 years) and required Critical Care or Trauma course	2 - 5 years	
Myanmar	YES	Cardiovascular Surgery<br>General Surgery<br>Maxillofacial Surgery<br>Neurosurgery<br>Otorhinolaryngology<br>Orthopedic Surgery<br>Pediatric Surgery<br>Plastic Surgery<br>Thoracic Surgery<br>Urology	Not reported.	2-3 years	

Nepal	YES	General Surgery<br>Otorhinolaryngology<br>Orthopedics and Trauma Surgery	Not reported.	3 years	
Nicaragua	None found.	NA	NA	NA	
Niger	YES	General Surgery<br>Surgery	Trauma competencies and placements required for all specialties (1 - 3 years) and required Critical Care or Trauma course	2 - 5 years	COSECSA
Nigeria	YES	Cardiothoracic Surgery<br>General Surgery<br>Neurosurgery<br>Orthopedics and Traumatology<br>Pediatric Surgery<br>Plastic Surgery and Burns Surgery<br>Urology	6 months trauma training for all programs with additional training in speciality-specific trauma scenarios.	3 - 8 years	WACS / ACS
Pakistan	YES	Cardiac Surgery<br>General Surgery<br>Neurosurgery<br>Orthopedic Surgery<br>Otorhinolaryngology<br>Pediatric Surgery<br>Plastic Surgery<br>Thoracic Surgery<br>Urology	Required competencies in specialty-specific trauma scenarios. Requirement of completion of ATLS workshop.	4 - 5 years	ACS / Arab Boards
Papua New Guinea	None found.	NA	NA	NA	
Philippines	YES	General Surgery<br>Neurosurgery<br>Otolaryngology<br>Orthopedic Surgery<br>Orthopedic Trauma Surgery<br>Pediatric Surgery<br>Plastic Reconstructive and Aesthetic Surgery<br>Thoracic and Cardiovascular Surgery<br>Trauma Surgery<br>Urology	Required competencies in specialty-specific trauma scenarios. Provision of Basic Emergency Skills in Trauma (BEST), Basic Evaluation and Training in Trauma and Emergency Response (BETTER) or ATLS courses for surgical residents, fellows and general medical practitioners.	Minimum 3 years	ACS
Rwanda	YES	General Surgery<br>Neurology<br>Orthopedic Surgery<br>Otorhinolaryngology<br>Plastic Surgery<br>Surgery<br>Urology	Trauma competencies and placements required for all specialties (1 - 3 years) and required Critical Care or Trauma course	2 - 5 years	COSECSA
São Tomé and Principe	None found.	NA	NA	NA	

Senegal	YES	Cardiothoracic Surgery<br>General Surgery<br>Neurosurgery	6 months trauma training for all programs with additional training in speciality-specific trauma scenarios.	5 - 8 years	WACS
Sierra Leone	YES	General Surgery<br>Surgery<br>Urology	6 months trauma training for all programs with additional training in speciality-specific trauma scenarios.	5 - 8 years	WACS
Solomon Islands	None found.	NA	NA	NA	
Somalia	YES	General Surgery<br>Surgery	Trauma competencies and placements required for all specialties (1 - 3 years) and required Critical Care or Trauma course	2 - 5 years	COSECSA
South Sudan	None found.	NA	NA	NA	
Sri Lanka	YES	Cardiothoracic<br>Gastrointestinal Surgery<br>Neurosurgery<br>Pediatric Surgery<br>Surgery<br>Thoracic Surgery<br>Urology	Required competencies in specialty-specific trauma scenarios.	3 - 4 years	
Sudan	YES	General Surgery<br>Neurosurgery<br>Orthopedic Surgery<br>Otorhinolaryngology<br>Pediatric Surgery<br>Plastic Surgery<br>Surgery<br>Urology	Trauma competencies and placements required for all specialties (1 - 3 years) and required Critical Care or Trauma course	2 - 5 years	COSECSA / Arab Boards
Syrian Arab Republic	YES	General Surgery<br>Neurosurgery<br>Orthopedic Surgery,<br>Otorhinolaryngology<br>Pediatric Surgery<br>Urology	Required competencies in specialty-specific trauma scenarios.	Not reported.	Arab Boards
Tajikistan	YES	Cardiac Surgery<br>General Surgery<br>Maxillofacial Surgery<br>Neurosurgery<br>Otorhinolaryngology<br>Pediatric Surgery<br>Plastic and Reconstructive Surgery<br>Thoracic Surgery<br>Traumatology and Orthopedics<br>Urology	Not reported.	2 years	

Tanzania	YES	General Surgery<br>Neurosurgery<br>Pediatric Orthopedic Surgery<br>Orthopedic Surgery<br>Surgery<br>Urology	Trauma competencies and placements required for all specialties (1 - 3 years) and required Critical Care or Trauma course	2 - 5 years	COSECSA
Timor-Leste	None found.	NA	NA	NA	
Togo	None found.	NA	NA	NA	
Tunisia	YES	Cardiovascular Surgery<br>General Surgery<br>Maxillo-Facial Surgery<br>Neurosurgery<br>Orthopedics and Traumatology<br>Otorhinolaryngology<br>Pediatric Surgery<br>Plastic Surgery<br>Thoracic Surgery<br>Urology	Required competencies in specialty-specific trauma scenarios. Generalists can also take part in additional injury management training or military medicine.	4 - 5 years	Arab Boards
Uganda	YES	General Surgery<br>Neurosurgery<br>Orthopedic Surgery<br>Pediatric Surgery<br>Plastic Surgery<br>Surgery<br>Urology	Trauma competencies and placements required for all specialties (1 - 3 years) and required Critical Care or Trauma course	2 - 5 years	COSECSA
Ukraine	YES	General Surgery<br>Orthopedics and Traumatology	Not reported.	Minimum 3 years	
Uzbekistan	YES	General Surgery<br>Maxillofacial Surgery<br>Pediatric Surgery<br>Plastic Surgery<br>Traumatology and Orthopedics<br>Urology	Not reported.	2 - 4 years	
Vanuatu	None found.	NA	NA	NA	
Vietnam	YES	Surgery	Not reported.	Not reported.	
West Bank and Gaza	YES	General Surgery<br>Neurosurgery<br>Orthopedic Surgery<br>Otorhinolaryngology<br>Urology	Not reported.	Not reported.	Arab Boards
Yemen, Rep.	YES	General Surgery	Not reported.	Not reported.	Arab Boards

Zambia	YES	General Surgery<br>Neurosurgery<br>Orthopedic Surgery<br>Otorhinolaryngology<br>Pediatric Surgery<br>Plastic Surgery<br>Surgery<br>Urology	Trauma competencies and placements required for all specialties (1 - 3 years) and required Critical Care or Trauma course	2 - 5 years	COSECSA
Zimbabwe	YES	Cardiothoracic Surgery<br>General Surgery<br>Orthopedic Surgery<br>Otorhinolaryngology<br>Pediatric Surgery<br>Plastic Surgery<br>Surgery<br>Urology	Trauma competencies and placements required for all specialties (1 - 3 years) and required Critical Care or Trauma course	2 - 5 years	COSECSA

ACS: American College of Surgeons; COSECSA: College of Surgeons of East, Central and South Africa; WACS: West African College of Surgeons
